# A Case Report of a Textiloma Causing Small Bowel Obstruction Two Years Following Open Appendectomy

**DOI:** 10.7759/cureus.98032

**Published:** 2025-11-28

**Authors:** Svetlana Ciocarlan, Raluca Belchita, Dimitrios Tsironis

**Affiliations:** 1 Department of General Surgery, St George’s University Hospitals NHS Foundation Trust, London, GBR; 2 Department of Upper Gastrointestinal Surgery, St George’s University Hospitals NHS Foundation Trust, London, GBR

**Keywords:** appendicectomy, case report, foreign body, laparoscopy, retained surgical item, small bowel obstruction, textiloma

## Abstract

Patients with retained surgical gauze in the abdomen can present with various clinical symptoms, including bowel obstruction. While typically occurring in the early postoperative period, such complications can also manifest months or even years after the original surgery, necessitating emergency intervention. A laparoscopic approach should be considered for cases of small bowel obstruction caused by a foreign body, with careful preoperative planning to reduce recovery time and length of hospital stay.

We report a case involving a 29-year-old man with a history of open appendicectomy during the COVID-19 pandemic in Brazil. He presented with symptoms of small bowel obstruction. Imaging revealed a radio-opaque object suggestive of retained surgical material. Emergency diagnostic laparoscopy revealed a retained gauze encapsulated within a cystic structure causing a closed-loop obstruction. The foreign body was successfully removed laparoscopically without bowel resection. This case highlights the importance of awareness and preparedness for late-presenting surgical complications.

## Introduction

Retained foreign bodies, such as surgical swabs, can present with a variety of symptoms, both early and late after surgery. While adhesions are a common cause of postoperative bowel obstruction, a retained foreign body represents a rare but significant etiology requiring surgical intervention. Radiological imaging, particularly CT, plays a critical role in diagnosis and surgical planning.

We report a rare case of an encapsulated retained surgical gauze causing extrinsic intestinal obstruction, which was successfully managed using a laparoscopic approach. Only a few such cases have been reported in the literature.

## Case presentation

A 29-year-old man who underwent an open appendicectomy two years prior in Brazil presented with a three-day history of generalized abdominal pain and multiple episodes of vomiting. Physical examination revealed abdominal distension and tenderness, predominantly in the right lower quadrant. A well-healed Lanz incision was noted without signs of infection.

**Figure 1:**
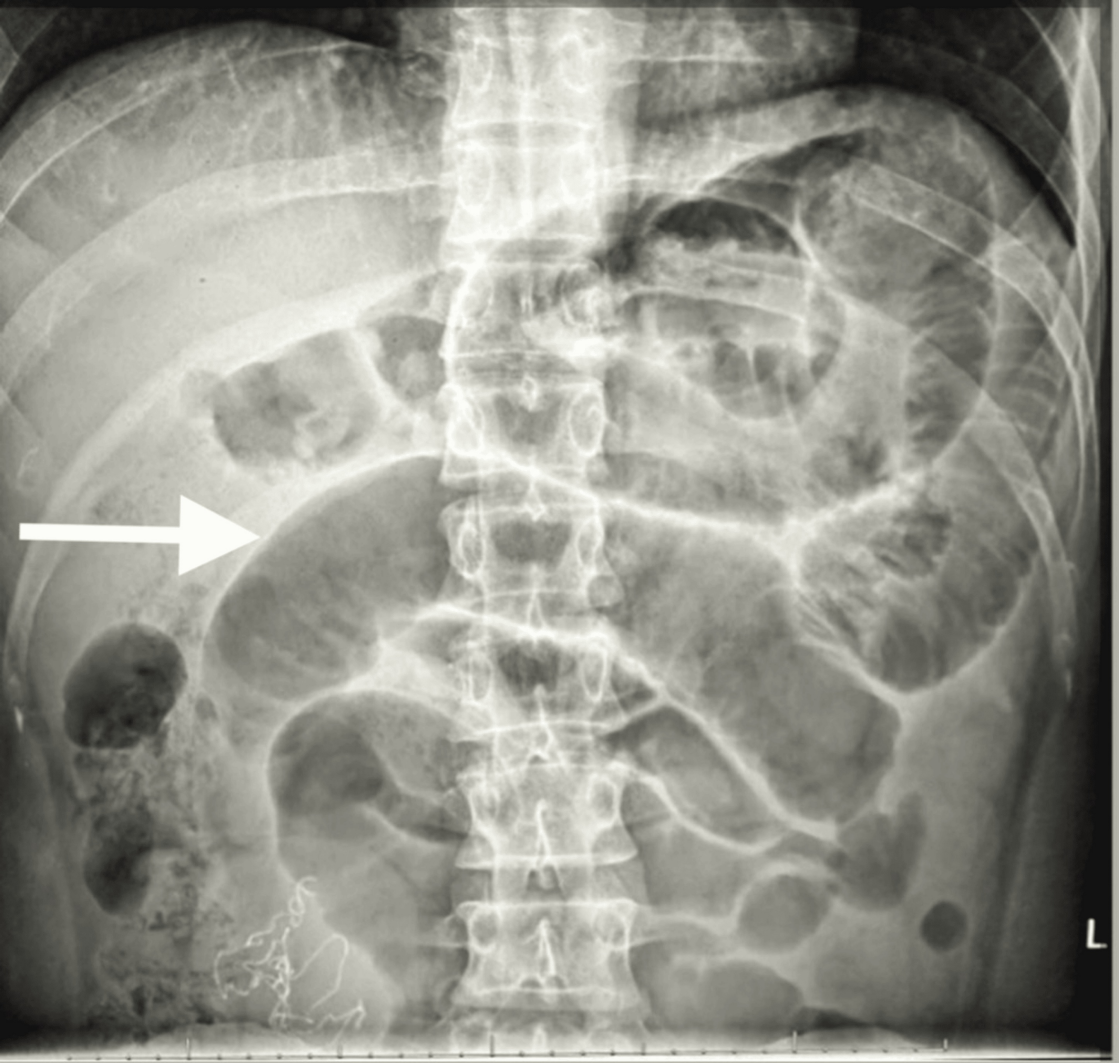
Plain abdominal X-ray demonstrating dilated loops of small bowel with multiple air-fluid levels, suggestive of mechanical small bowel obstruction

**Figure 2:**
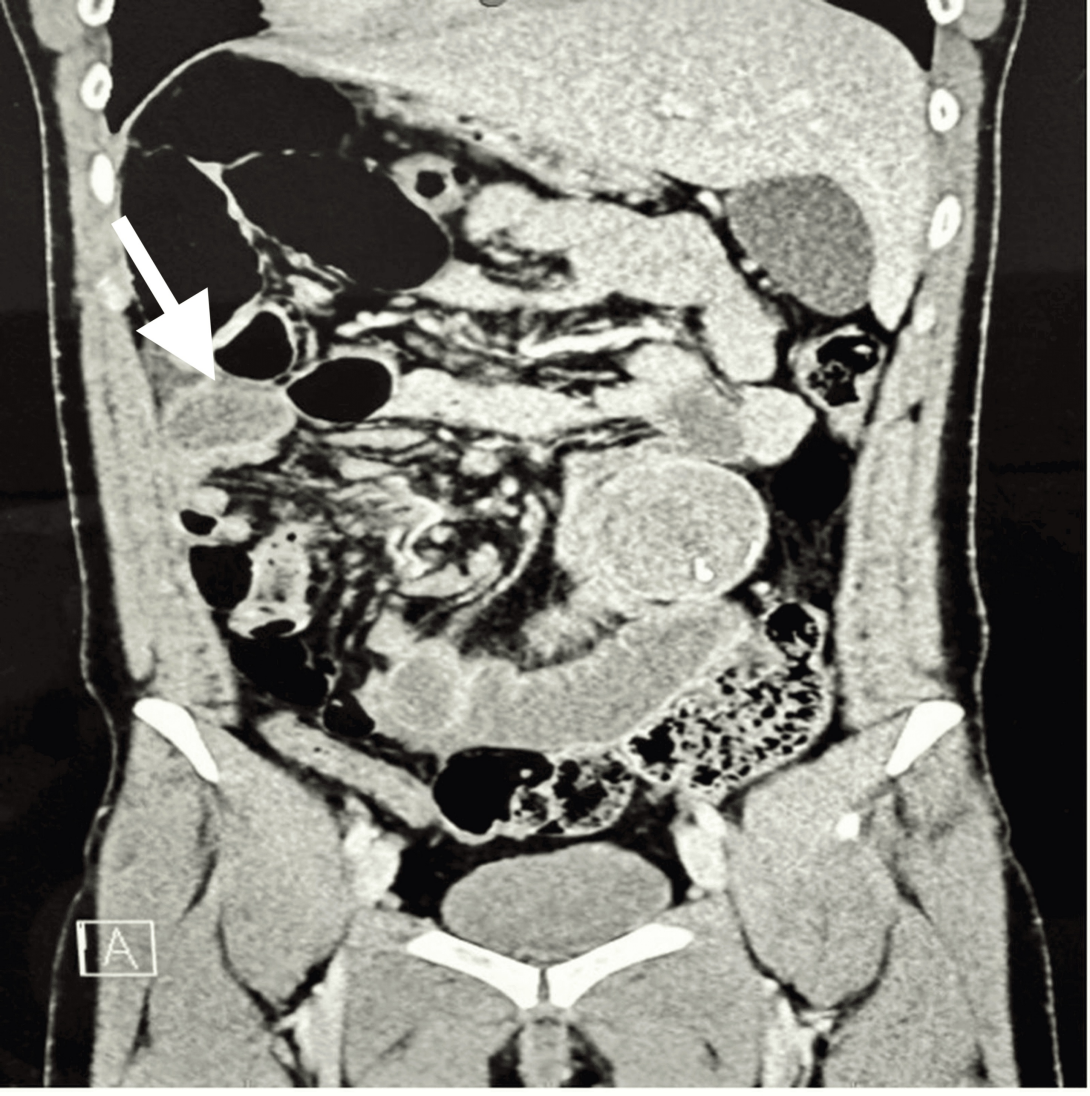
Coronal view of abdominal CT showing a well-defined encapsulated mass with surrounding small bowel loops, consistent with closed-loop obstruction due to a retained surgical item

Laboratory investigations showed mild leukocytosis (WBC: 13.4 × 10³/mm³; normal range: 4.0-11.0 × 10³/mm³) and low bicarbonate levels (21 mmol/L; normal range: 22-28 mmol/L), with other biochemical parameters within normal limits. Plain abdominal radiography and contrast-enhanced CT (Figures 1, 2) revealed a walled-off collection containing a radiopaque object, indicative of retained surgical material, causing small bowel obstruction.

**Figure 3:**
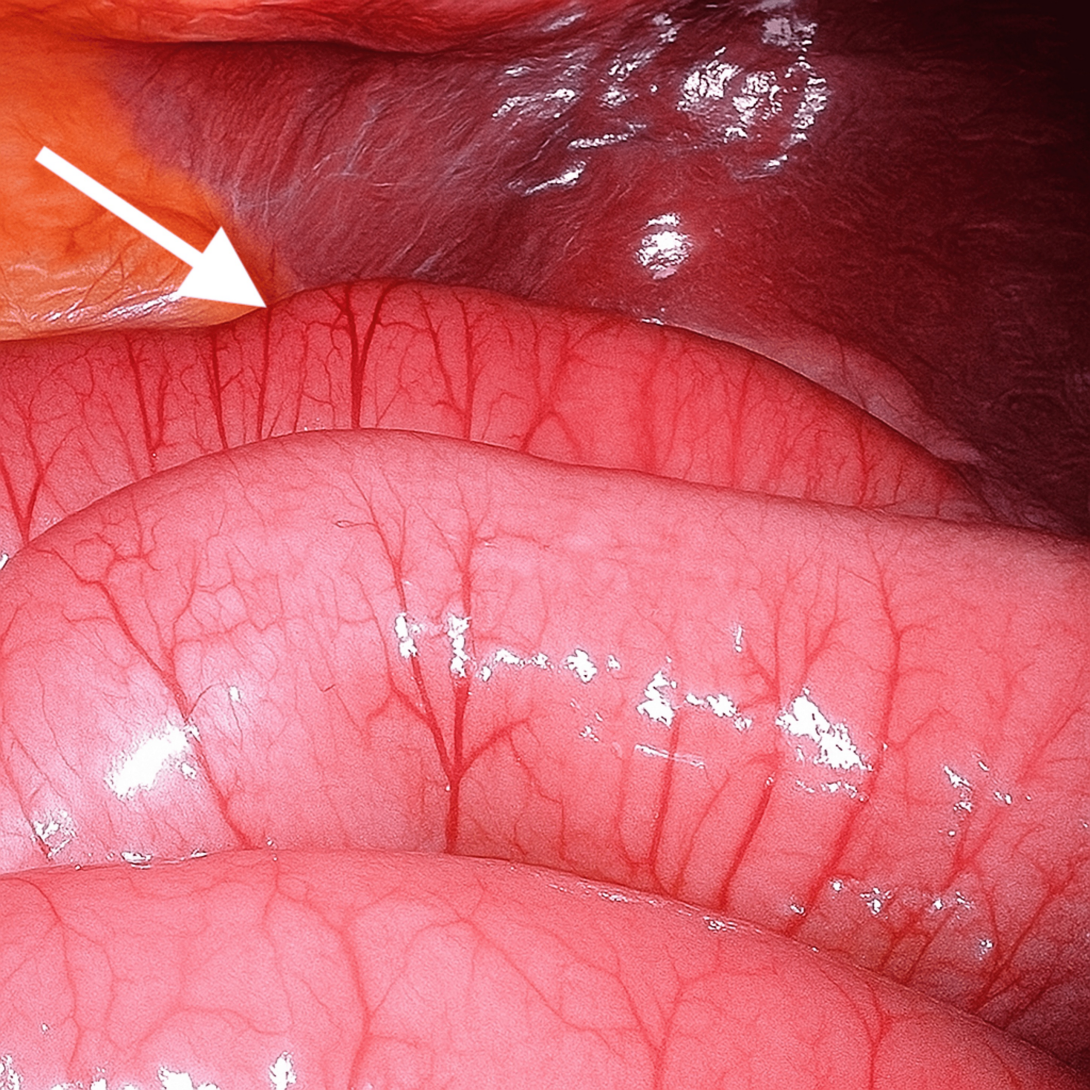
Laparoscopic view of dilated small bowel loops and adjacent inflamed omentum

**Figure 4:**
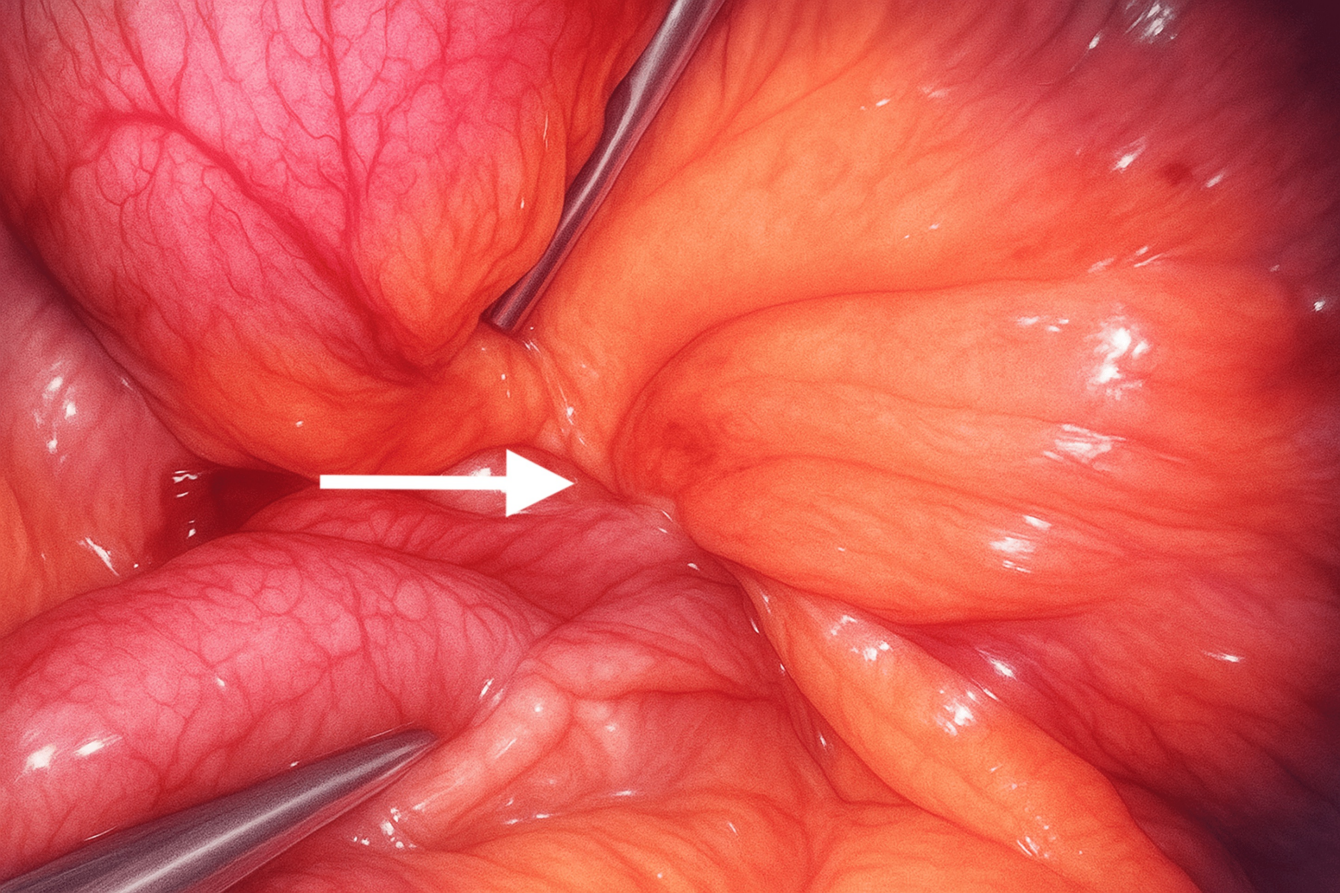
Laparoscopic view demonstrating a closed-loop small bowel obstruction with dilated and congested loops adhered to an inflammatory mass

**Figure 5:**
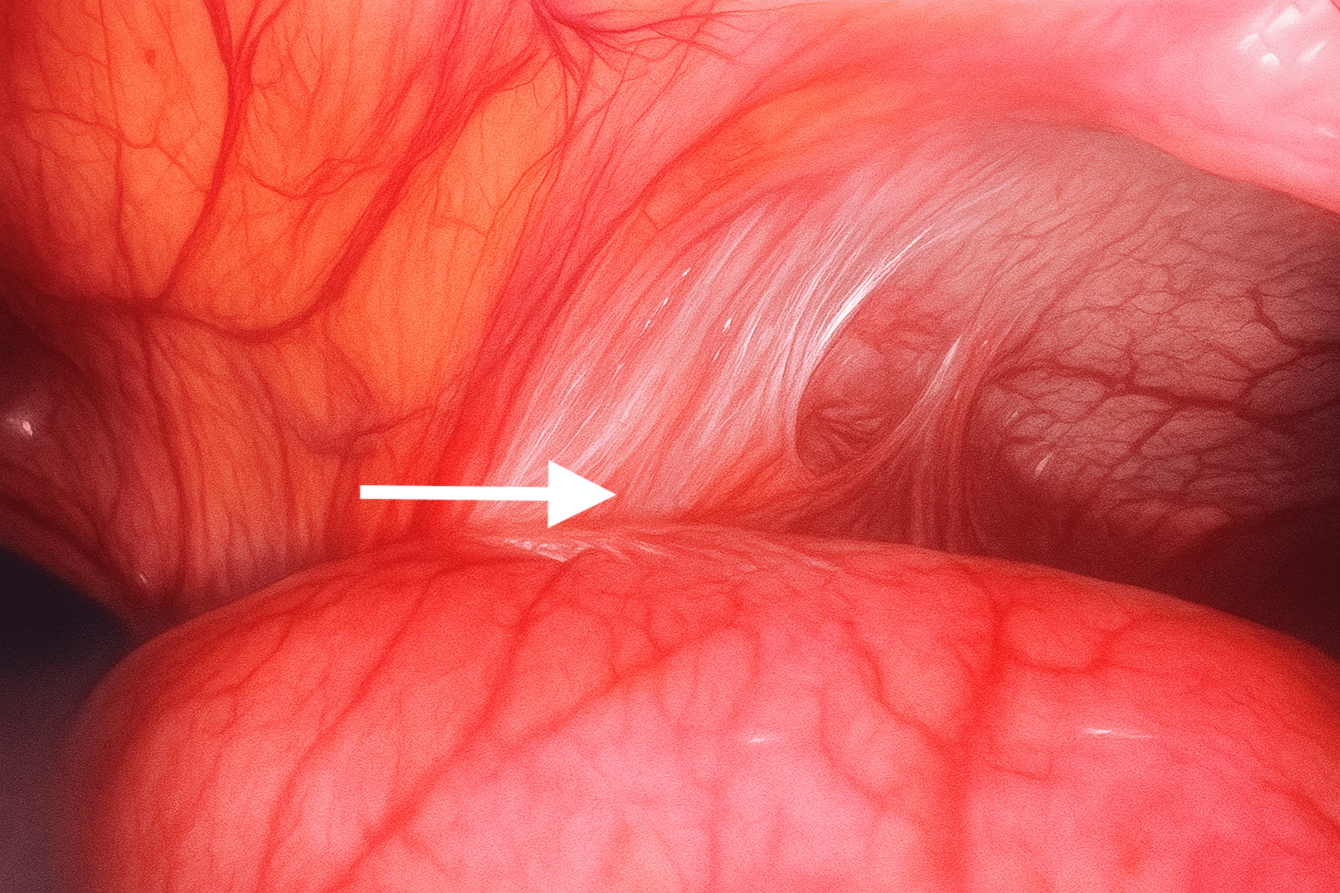
Intraoperative laparoscopic image showing dilated small bowel loops adherent to the omentum and surrounding inflammatory tissue

**Figure 6:**
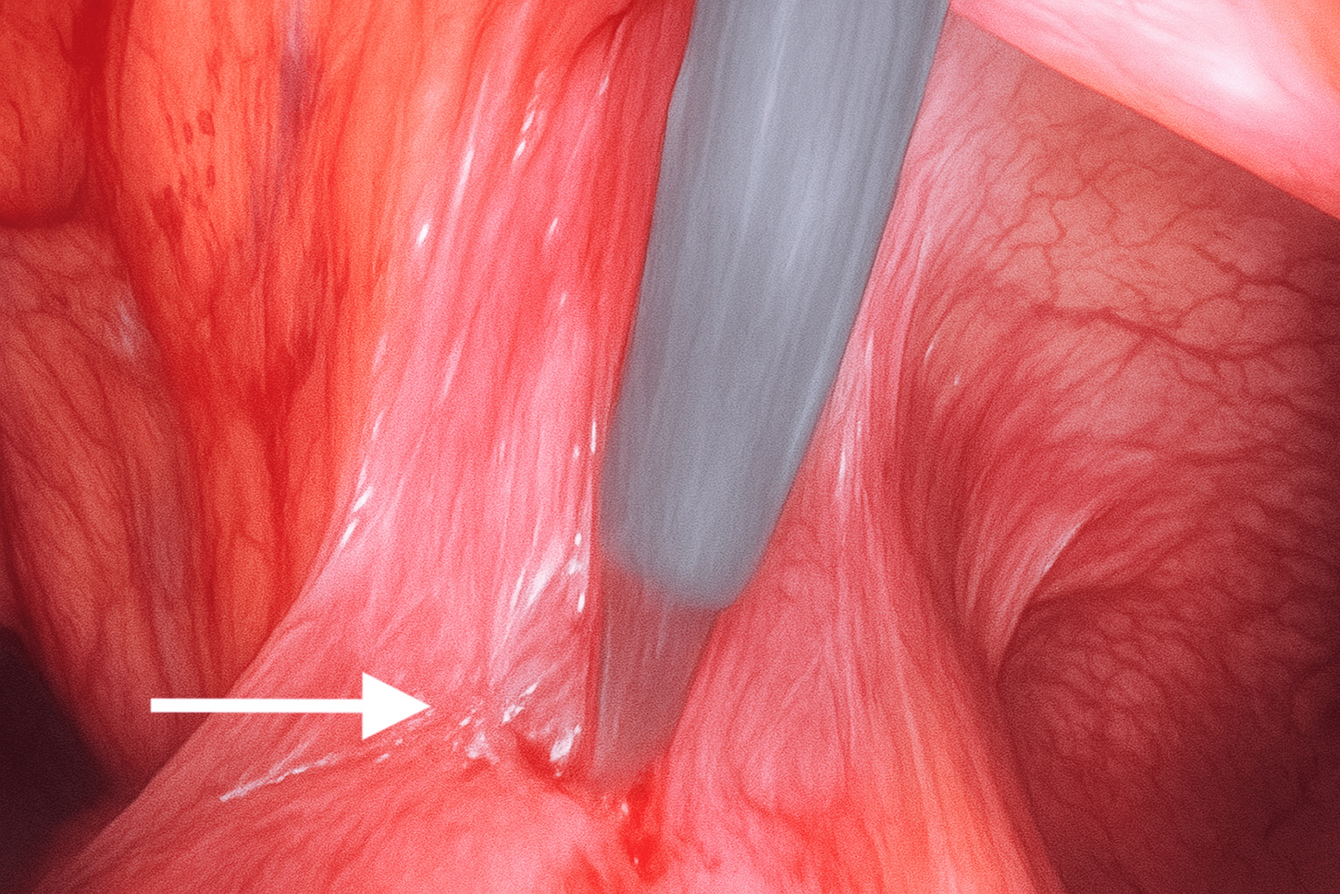
Division of the adhesive band connecting the small bowel loop to the capsule using laparoscopic scissors

The patient underwent emergency diagnostic laparoscopy. Intraoperatively, a closed-loop obstruction (Figures 3, 4) was observed, with small bowel loops adherent to a large cystic mass containing an encapsulated swab surrounded by pus and partially covered by omentum (Figures 5, 6). Dense adhesions were present in the area, with one band adhesion between the small bowel and the capsule contributing to the obstruction (Figure 4).

**Figure 7:**
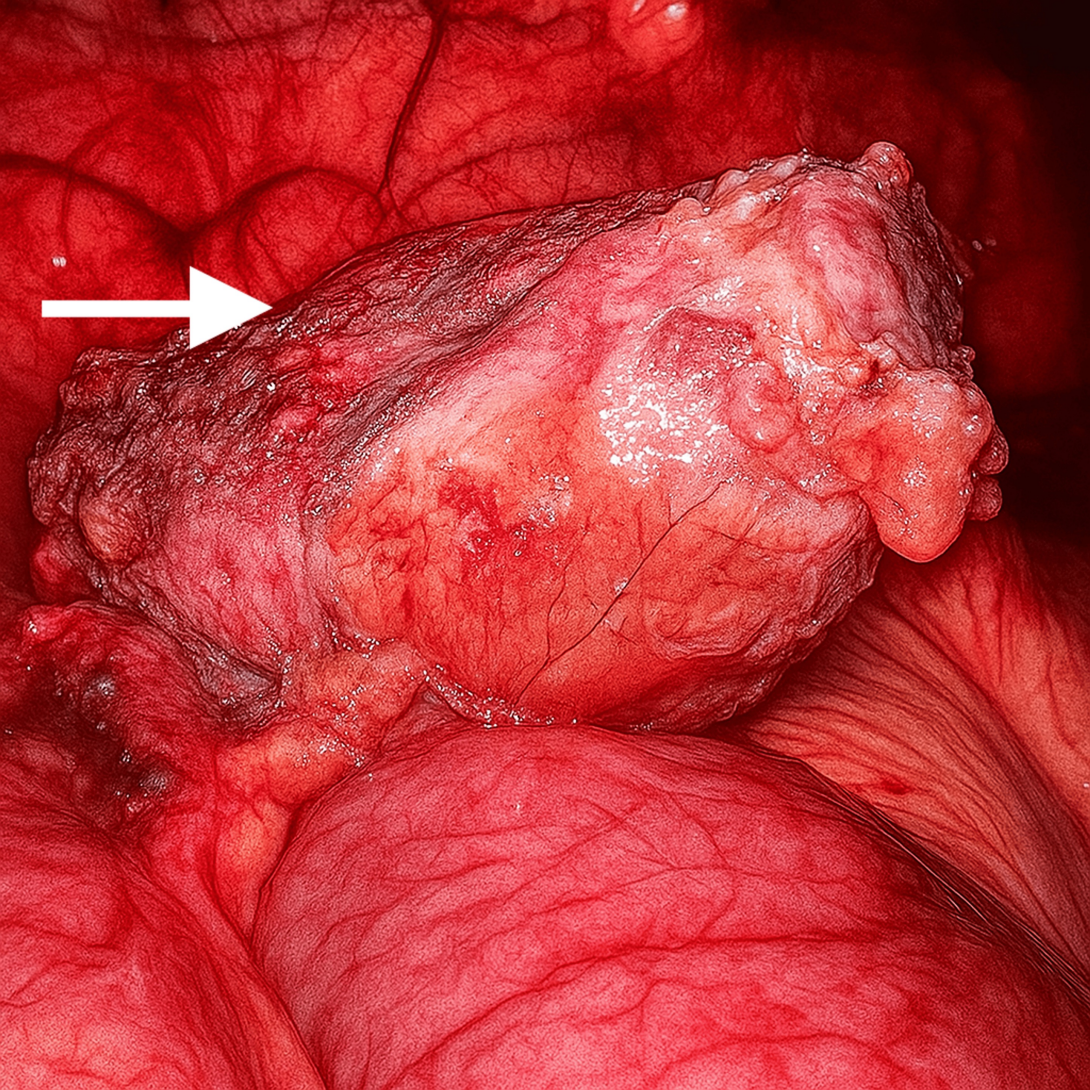
Laparoscopic view of the encapsulated foreign body (textiloma) surrounded by inflamed tissue and omental adhesions

**Figure 8:**
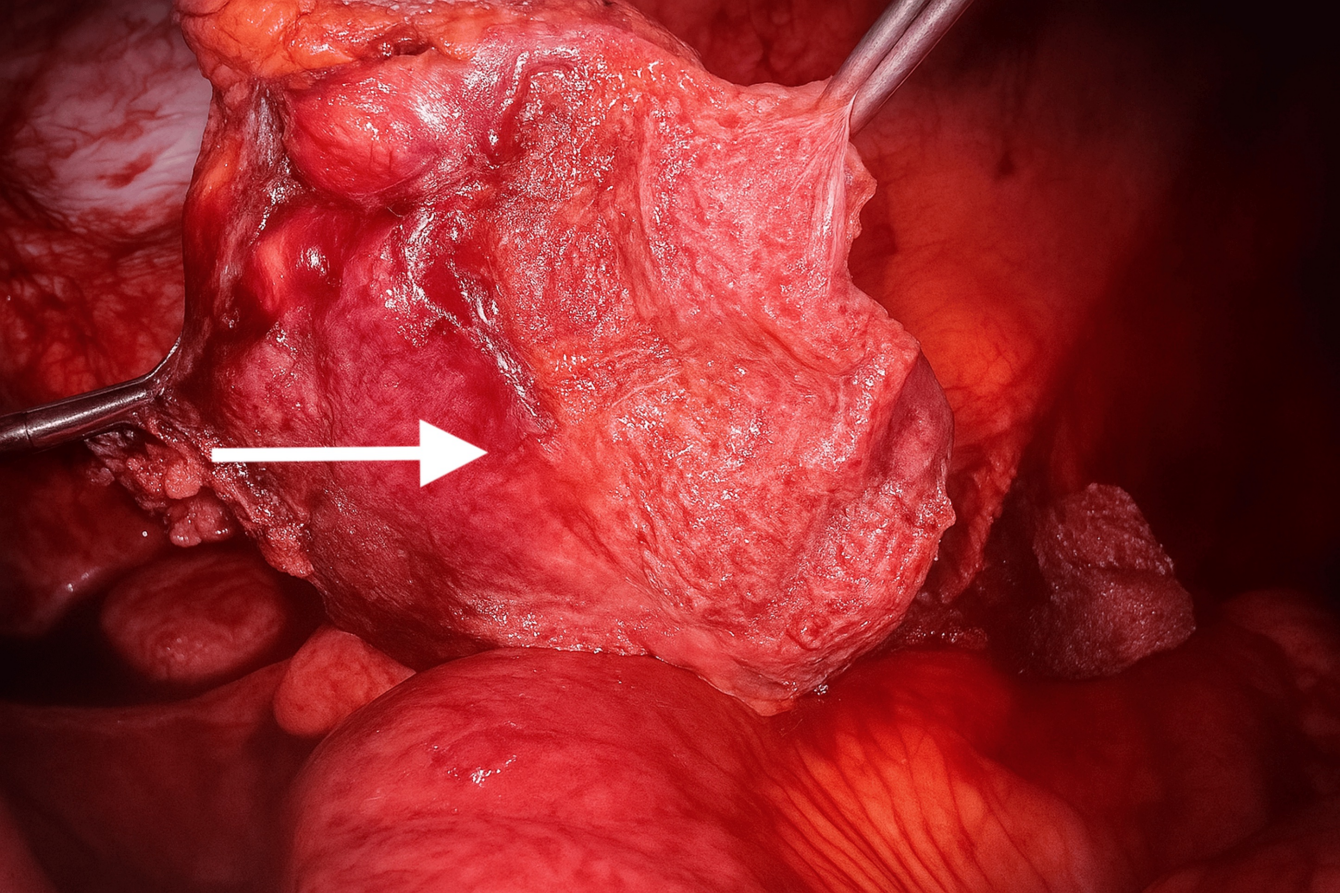
Dissection and mobilization of the encapsulated textiloma using laparoscopic instruments

**Figure 9:**
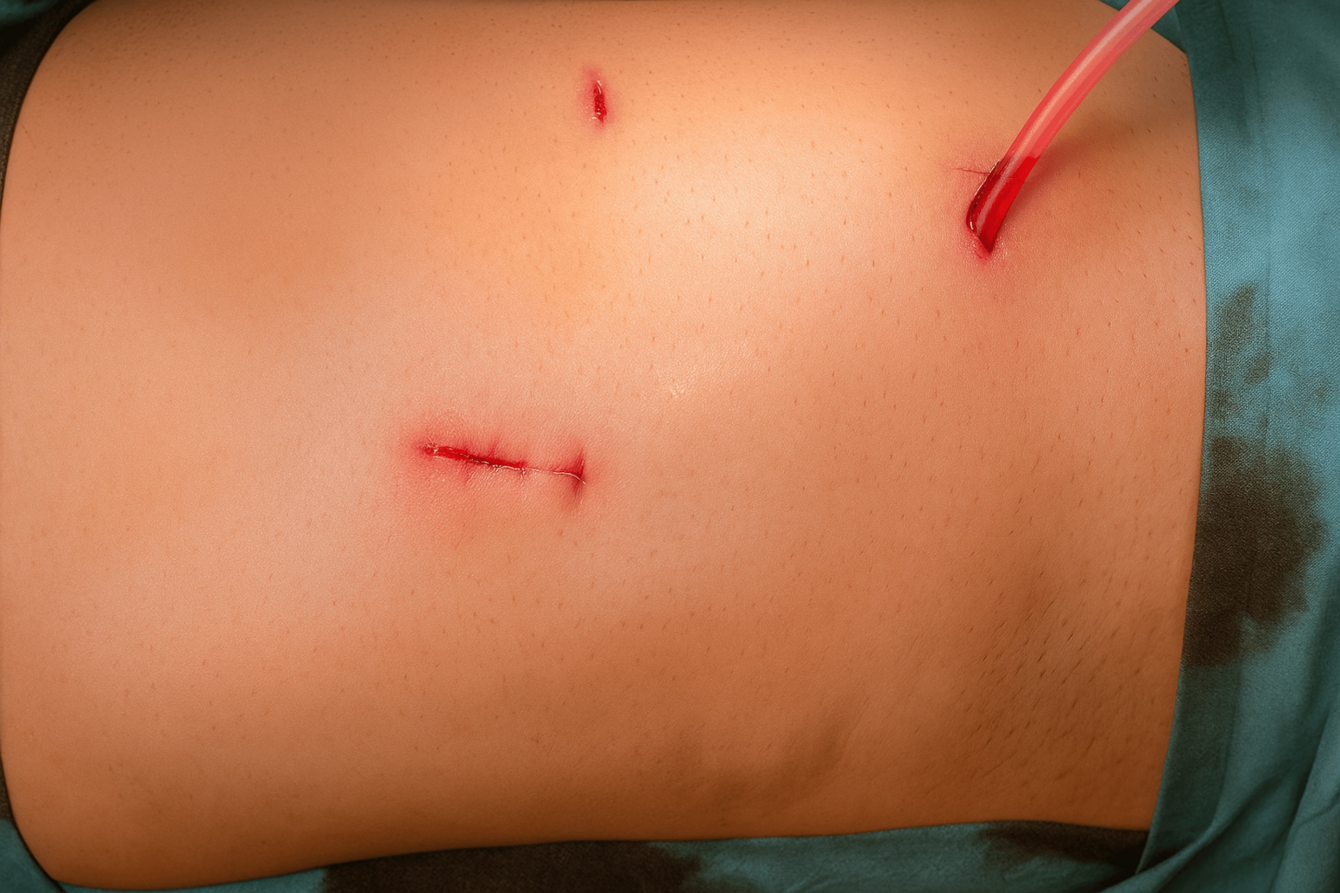
Immediate postoperative appearance of the abdomen following laparoscopic removal of the retained textiloma

**Figure 10:**
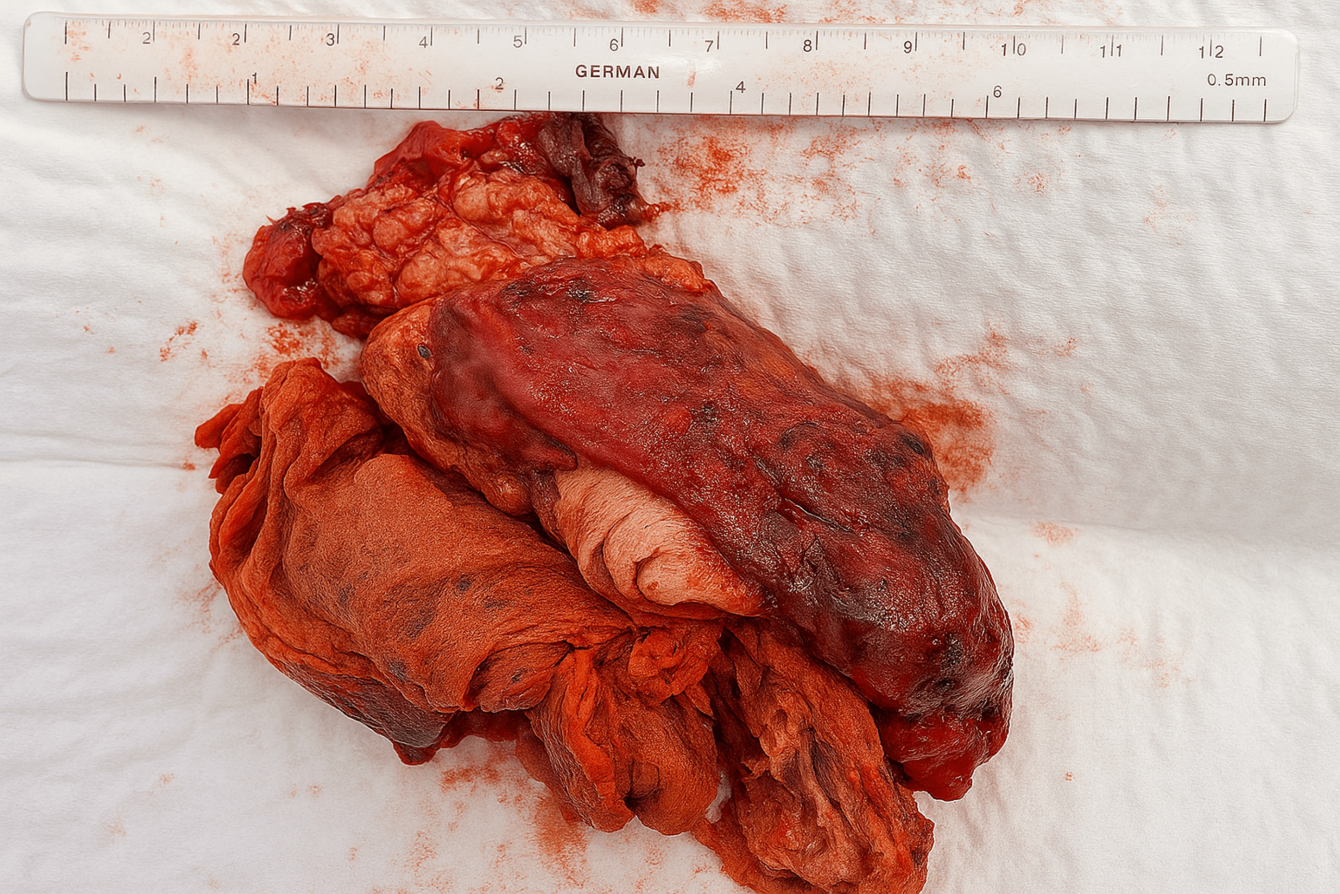
Gross specimen of the retained surgical gauze (textiloma) removed during laparoscopic surgery

Adhesiolysis was performed, and the band adhesion was divided to release the bowel (Figures 5, 6). The cystic capsule was perforated to aspirate the pus, and the encapsulated swab was carefully dissected from the abdominal wall, small bowel, and omentum (Figures 7-9). Figure 9 shows that a drain was placed in the pelvis. The excised specimen is shown in Figure 10.

**Figure 11:**
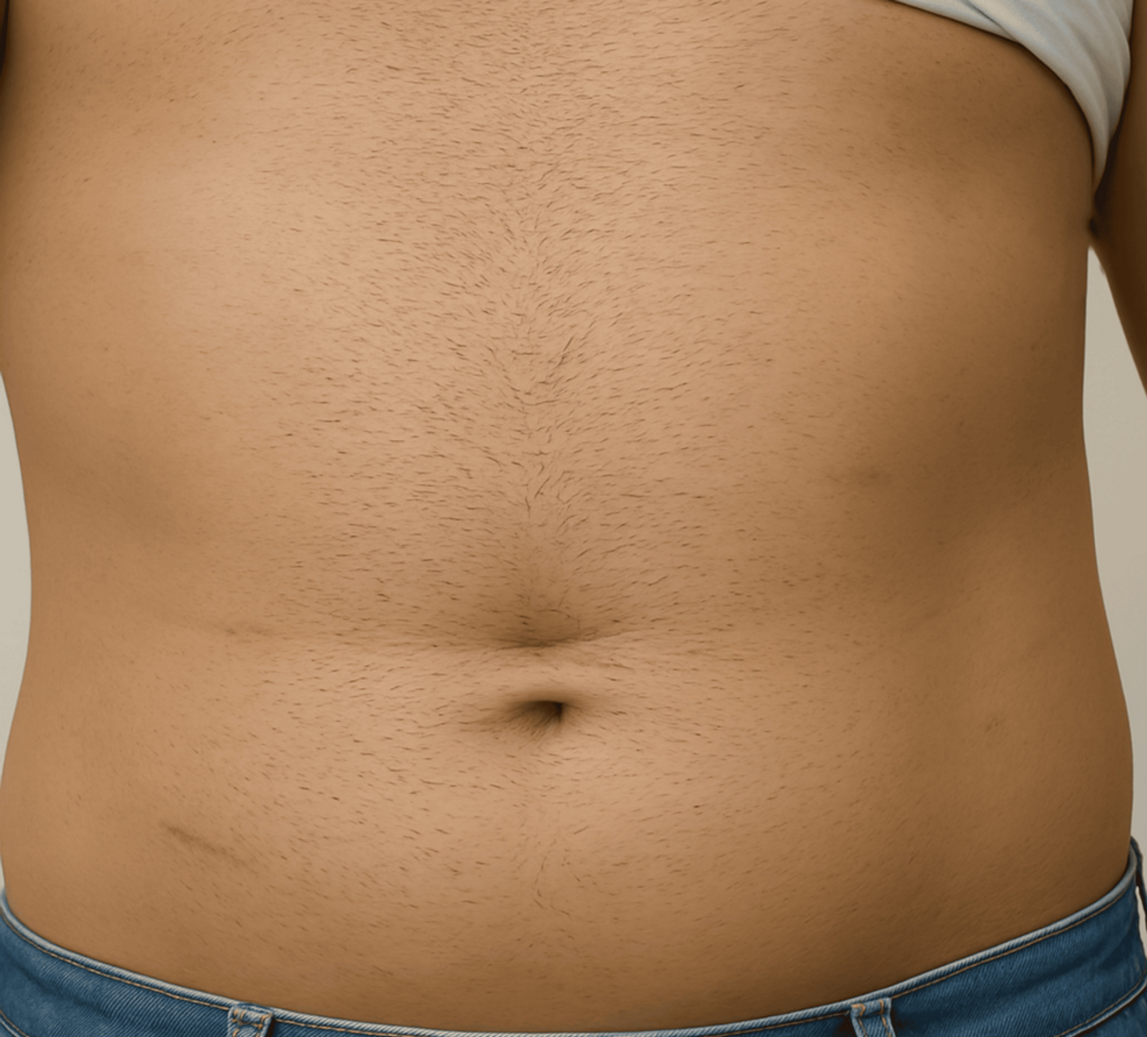
Follow-up image showing healed port sites and absence of complications three months postoperatively

The patient recovered well postoperatively, and the drain was removed on the fifth postoperative day. Oral intake was resumed, and the patient was discharged. Histological analysis confirmed an abscess wall with a foreign body granulomatous reaction to gauze. At three-month follow-up, the patient’s port sites had healed well (Figure 11).

## Discussion

Retained surgical items (RSIs) are rare but serious complications with an incidence of 0.3-1.0 per 1,000 abdominal operations [1]. The WHO surgical checklist [2] is one such protocol with proven benefits. As classified by the American College of Surgeons, retained materials are considered preventable never events [3]. These retained items may cause significant morbidity and legal consequences, including abscesses, fistulas, and bowel obstruction. Known risk factors include emergency procedures, long or complex surgeries, unexpected intraoperative changes, and inexperience among surgical staff. We report a rare case of an encapsulated retained surgical gauze causing extrinsic intestinal obstruction, which was successfully managed using a laparoscopic approach.

This case is significant because the gauze remained undetected for over two years, highlighting the potential for delayed complications. Similar delays in presentation have been reported, ranging from days to decades postoperatively [1,4]. In our case, the foreign body had become encapsulated in a fibrous cystic structure, which contributed to the obstruction. Histological findings confirmed chronic inflammation with granuloma formation, a common finding in long-standing RSIs [5].

Risk factors for RSIs are well established: emergency procedures, high body mass index, unplanned intraoperative changes, and communication failures [3,6]. Our case occurred during the COVID-19 pandemic, a time of resource strain and altered staffing, conditions known to increase surgical error risk.

Importantly, we emphasize that a laparoscopic approach is not contraindicated in these cases. Laparoscopy allowed effective diagnosis, safe dissection, and complete removal of the retained gauze without the need for bowel resection. Literature suggests laparoscopy is feasible in select stable patients and may reduce morbidity, hospital stay, and recovery time compared to open procedures [4,6].

This case reinforces the essential role of intraoperative counts, radiopaque markers, and surgical checklists [2]. Institutional adherence to WHO safety protocols is critical to prevent such avoidable complications. Furthermore, when suspicion arises, timely imaging and early surgical intervention are paramount.

## Conclusions

We presented a rare case of symptomatic retained surgical gauze causing small bowel obstruction two years following appendicectomy. The condition was successfully managed laparoscopically, avoiding bowel resection. This case reinforces the need for vigilance during surgery and highlights the effectiveness of a laparoscopic approach when feasible.

## References

[1] Wan W, Le T, Riskin L, Macario A (2009). Improving safety in the operating room: a systematic literature review of retained surgical sponges. Curr Opin Anaesthesiol.

[2] American College of Surgeons (ACS) Committee on Perioperative Care (2016). Revised statement on the prevention of unintentionally retained surgical items after surgery. Bull Am Coll Surg.

[3] Lincourt AE, Harrell A, Cristiano J, Sechrist C, Kercher K, Heniford BT (2007). Retained foreign bodies after surgery. J Surg Res.

[4] Gawande AA, Studdert DM, Orav EJ, Brennan TA, Zinner MJ (2003). Risk factors for retained instruments and sponges after surgery. N Engl J Med.

[5] (2025). World Health Organization. WHO surgical safety checklist and implementation manual. https://iris.who.int/handle/10665/70046.

[6] (2025). Gossypiboma. https://radiopaedia.org/articles/gossypiboma.

